# Determinants of Inequalities in the Exposure to and Adoption of Multiple Health Risk Behaviors among Brazilian Adolescents, 2009–2019

**DOI:** 10.3390/ejihpe14070135

**Published:** 2024-07-11

**Authors:** Mariana Rebello Haddad, Flavia Mori Sarti

**Affiliations:** 1 Biomedical Center, Department of Integrated Education in Health, Federal University of Espirito Santo, Vitória 29047-185, ES, Brazil; 2 School of Arts, Sciences and Humanities, University of Sao Paulo, Sao Paulo 03828-000, SP, Brazil; flamori@usp.br

**Keywords:** multiple risk behaviors, social determinants of health, health behavior, adolescence, inequalities

## Abstract

The occurrence of multiple risk behaviors among adolescents imposes challenges in the context of public policies of health, particularly in low- and middle-income countries. Evidence on the conditions leading to the exposure to and adoption of multiple risk behaviors allows the identification of vulnerable groups of adolescents, and may support the proposition of targeted strategies directed to individuals at risk. Therefore, the aim of this study was to perform a quantitative analysis to identify recent trends in the exposure to and adoption of multiple health risk behaviors among Brazilian adolescents, highlighting individual-, household-, and school-level characteristics linked to inequalities among social groups. The analysis was based on cross-sectional data from the National Student Health Survey (PeNSE), conducted by the Brazilian Institute for Geography and Statistics in 2009, 2012, 2015, and 2019. The trends in the occurrence of multiple risk behaviors among adolescents were estimated according to social strata, allowing the calculation of concentration indexes and their disaggregation into major determinants of inequalities in the exposure and adoption of risk behaviors. The analyses were conducted using a complex survey design to allow representativeness at the population level. The results showed a rise in the incidence of multiple risk behaviors among youngsters in Brazil from 2009 to 2019. Factors influencing inequalities in the exposure to multiple risk behaviors were socioeconomic status and the characteristics of the household and school environments, whilst the adoption of multiple risk behaviors was also influenced by early exposure to multiple risk behaviors. Furthermore, trends in inequalities in the exposure to and adoption of multiple risk behaviors showed an intensification from 2009 to 2019, being initially concentrated among wealthier adolescents, followed by a transition to higher incidence in the lower socioeconomic strata in 2012 and 2015, respectively. The findings underscore the role of support systems for adolescents at risk within the familial and school contexts, whereas strategies of public policies of health based on the strengthening of community ties may require improvements to tackle socioeconomic inequalities in the occurrence of risk behaviors among youngsters.

## 1. Introduction

Adolescence represents a challenging life stage linked to substantial changes in various dimensions of the daily life of individuals. The cognitive, emotional, and physical transformations experienced during adolescence comprise drivers for experimentation, exercising decision-making and autonomy, and rebellion against rules and authority figures. In addition, changes during adolescence may be triggers for negative feelings (e.g., insecurity, depression, and anxiety), contributing to the initiation of health risk behaviors compelled by peer pressure, upheaval, or a sense of inadequacy [1,2,3].

Furthermore, the social interactions established by the adolescents with their peers and their families or other individuals within the household and the school environments may influence the formation and the choices of youngsters, potentially enabling the exposure to and the maintenance of risk behaviors [4,5]. An integrative review of the literature synthesized the effects of social capital on the formation of health risk behaviors in adolescents from diverse countries, showing that positive family relationships, parents’ attention to the adolescents’ activities, and school quality may comprise important factors to minimize trends in health risk behaviors [6].

The promotion of a positive household environment through family support includes attention to daily activities and attendance at school, in addition to fostering healthy habits like family meals and leisure practices. On the contrary, a negative household environment may enable the ascendence of the influence of other social actors on adolescents’ choices, including peer pressure, opinions of friends, and the imitation of school colleagues and teachers, among others [7,8,9]. Similarly, experiences at school may be marked by the support of friends, colleagues, and teachers or situations of bullying and violence, characterizing positive or negative school environments with diverse effects on adolescents’ health risk behaviors, respectively [9].

Other elements that influence adolescents’ exposure to risk, including contextual attributes (i.e., economic crises, changes in public policies of health, and others), may represent challenges or opportunities to reduce the assimilation of risk behaviors during adolescence [10]. Strategies of public health based on strengthening community ties tend to prevent the consolidation of multiple health risk behaviors [11], particularly health programs to monitor families’ health through regular household visits of multidisciplinary health professionals’ teams with the participation of community health workers, like the Brazilian Family Health Strategy. Furthermore, the factors influencing the initiation of health risk behaviors during adolescence display complex interactions, which may encourage simultaneous experiences with diverse health risk behaviors, leading to the early co-occurrence of health risk behaviors [12,13,14,15,16,17,18].

The increase in the adoption of multiple risk behaviors among youngsters represents a worrying trend in recent decades [19,20], especially considering that health behaviors initiated during childhood and adolescence tend to persist throughout adulthood [3]. The exposure to and adoption of multiple risk behaviors compromise short- and long-term health outcomes of the population, including well-being and productivity, which may turn into a vicious cycle of disease, productivity loss, unemployment, and impoverishment among vulnerable individuals [10], especially in low- and middle-income countries characterized by considerable socioeconomic inequalities like Brazil.

The investigation of the conditions leading to the exposure and adoption of multiple risk behaviors among adolescents allows the identification of vulnerable groups and the proposition of strategies in public policies of health targeting individuals at risk. Yet, the major part of the studies on the exposure to and adoption of multiple health risk behaviors focuses on adolescents in high-income countries [6]. Evidence from the 2015 Youth Risk Behavior Surveillance System showed a high adoption of risk behaviors among US adolescents in the context of unhealthy eating patterns, unsafe sexual behavior, abusive alcohol consumption, and the use of tobacco [4].

The National Student Health Survey (PeNSE) was designed to monitor risk and protection factors to inform evidence-based decision-making processes in public policies directed to adolescents’ health in Brazil. The PeNSE has been conducted regularly every 3–4 years since 2009, allowing the assessment of the effectiveness of public health programs and campaigns targeting risk factors among youngsters [11,21]. Studies on health risk behaviors using data from the PeNSE usually focus on inequalities of the incidence of isolated behaviors [18], or the co-occurrence of risk behaviors among Brazilian adolescents [15,16,20]. However, there is a lack of evidence on trends and determinants of inequalities in the exposure to and adoption of multiple risk behaviors among Brazilian students throughout the period from 2009 to 2019.

Previous evidence showed an increase in the occurrence of unsafe sexual practices and violence and an increase in alcohol consumption in Brazilian adolescents until 2015, although there was a higher probability of co-occurrence of the use of cigarettes and alcohol consumption among youngsters in Brazil [20]. Nevertheless, there was an absence of studies investigating links between public policies of health and changes in inequalities in the occurrence of multiple risk behaviors during adolescence, including the use of cigarettes, the consumption of alcoholic beverages, interpersonal violence, sexual behavior, traffic practices, and the use of illicit drugs.

Therefore, the present study aimed to investigate the trends and determinants associated with inequalities in the exposure to and adoption of multiple health risk behaviors among Brazilian youngsters between 2009 and 2019, including the potential role of prevention performed by the Brazilian Health Family Strategy. The research focuses on the dissemination of evidence to support the design of public policies targeting vulnerable adolescents, considering that the publication of scientific evidence comprises a key element for evidence-based decision-making in the design, implementation, and evaluation of public policies of health in Brazil.

## 2. Materials and Methods

### 2.1. Study Design

The study focuses on the quantitative analysis of cross-sectional data from four editions of the National Student Health Survey (PeNSE), performed by the Brazilian Institute for Geography and Statistics (IBGE) through a partnership established with the Brazilian Ministry of Health. The survey is conducted every 3–4 years in Brazil since 2009, i.e., there are four editions currently available: 2009 (n = 63,411), 2012 (n = 61,145), 2015 (n = 51,135), and 2019 (n = 40,017).

The adolescents invited to participate in the four editions of the survey were students enrolled in public or private schools in Brazil, independently of their age. The first edition of PeNSE (2009) selected students enrolled in the last year of primary school (9th grade) in the 26 Brazilian state capitals and in the Federal District (Brasilia). The second edition of PeNSE (2012) included an additional subsample of interviews with students enrolled in schools out of state capitals clustered into the five Brazilian macro-regions. The third edition of PeNSE (2015) included an additional subsample of interviews with students enrolled in other school levels to allow comparison with the international survey of the Global School-Based Student Health Survey (GSHS). Finally, the fourth edition of PeNSE (2019) involved a sample of students enrolled from the later primary school (7th to 9th grade) to the secondary school (1st to 3rd year), being representative at population level.

The adolescents were selected using probabilistic sampling process in two stages (schools and classes within schools) for representativeness at the level of Brazilian state capitals. The sample design was based on information from the previous School Census available during survey planning (i.e., using data from 2007, 2010, 2013, and 2017, respectively) [22,23]. Considering the changes in the target population across the four editions of the survey, the present study focused on the analysis of data from subsamples that were directly comparable, according to information from the Brazilian Institute for Geography and Statistics, i.e., individuals with similar characteristics of the target population interviewed in 2009 [24,25].

The study was approved by the Brazilian National Research Ethics Commission (opinions #11,537; #16,805; #1,006,467; and #3,249,268), following the ethical principles of the Helsinki Declaration. The adolescents were invited to the research and signed electronic written consent before participating in the study. Parents or relatives were informed of the participation of the adolescents in the study [21,22,23,24,25]. The Brazilian Child and Adolescents Statute (Law 8,069/1990) grants autonomy to adolescents regarding the decision to participate in surveys with an observational design to support public policy decision-making; thus, parents were exempt from signing informed consent. The Brazilian National Research Ethics Commission prohibits approaching human subjects for participation in surveys and other studies in exchange for money, and therefore, neither the adolescents nor their parents or relatives received payment for participating in the survey.

### 2.2. Data

The datasets encompass individual-, household-, and school-level data collected by the IBGE through electronic questionnaires presented in portable devices to adolescents attending public and private schools. The National Student Health Survey (PeNSE) comprises part of the national efforts to monitor adolescents’ health, risk factors, and health-related behaviors. The selection of variables from PeNSE datasets was based on the availability of information directly comparable across the four editions of the survey.

The PeNSE questionnaire encompasses subsections on sociodemographic, lifestyle, and health-related questions previously validated and adopted in other population-based surveys, including family and household characteristics, and food consumption patterns and practices; physical activity during physical education classes, transportation, and others; tobacco use, the consumption of alcoholic beverages, and the use of illicit drugs; security in the school and the streets; mental health; sexual experience; oral health and personal hygiene; the utilization of health services; and body image.

Questions on health risk behaviors were based on the questionnaire of the Youth Risk Behavior Surveillance System, translated, adapted, and validated for the Brazilian population [21,26]. Part of the questions included in each subsection of the questionnaire changed according to the edition of the survey; however, several questions were maintained, allowing the comparison of variables throughout the four editions. The timeframe in the questions regarding risk behaviors was defined by the Brazilian Institute for Geography and Statistics based on previously validated questionnaires.

This study focused on the analysis of inequalities in the exposure to and adoption of multiple risk behaviors among Brazilian students, including only variables that were directly comparable in the four editions of PeNSE, i.e., questions that were similar in 2009, 2012, 2015, and 2019. The outcome variables investigated were two composite indicators based on the estimation of the proportion of adolescents declaring multiple health risk behaviors: (1) exposure to multiple health risk behaviors, and (2) adoption of multiple health risk behaviors. The risk behaviors investigated corresponded to the use of cigarettes, the consumption of alcoholic beverages, the use of illicit drugs, sexual behavior, involvement in episodes of violence, traffic practices, and reckless driving.

The emission of driver permits, and the sale or utilization of licit drugs (e.g., cigarettes and alcoholic beverages) are forbidden for individuals under 18 years of age, and the sale or use of any illicit drugs (e.g., marijuana, cocaine, and crack) is prohibited in Brazil. Therefore, driving and the consumption of cigarettes, alcoholic beverages, and illicit drugs were considered risky behaviors in the study. Concerning sexual behavior, the age of consent in Brazil is 18 years old, and therefore adolescents reporting an initiation of sexual experiences and the absence of the use of condoms during sexual relations were considered at risk.

Regarding violence, two risk behaviors were considered in the context of the present study: involvement in fights with firearms, and involvement in fights with other types of weapons (knives, blunt objects, or others). Referring to traffic practices, violations of the Brazilian laws regarding the requirements of the use of seatbelts in cars or helmets in motorcycles, and of the prohibition of driving under the influence of alcohol were considered risk behaviors.

Thus, the outcome variable representing the exposure to multiple risk behaviors referred to the co-occurrence of three or more of the following experiences, according to the variables available in the survey: the use of cigarettes at least once in life, the consumption of alcoholic beverages at least once in life, the use of any illicit drug at least once in life, intercourse at least once in life, involvement in fights with firearms or other types of weapons in the last 30 days, the adoption of any unsafe practice in traffic during the last 30 days (the absence of a seatbelt in cars or a helmet in motorcycle rides, or riding a vehicle driven by a drunk driver), and driving without a permit at least once in the last 30 days. The cutoff point regarding the co-occurrence of three of more experiences was based on the requirement to establish a pattern of exposure to health risk behaviors, especially because 50% of the experiences were characterized as occurring once in the adolescent’s lifetime (the use of cigarettes, the consumption of alcoholic beverages, the use of illicit drugs, and sexual intercourse).

The outcome variable representing the adoption of multiple risk behaviors referred to the co-occurrence of two or more of the following high-risk behaviors according to the variables available in the survey: the use of cigarettes for more than 6 days in the last 30 days, the abusive consumption of alcohol more than three times in life, the use of illicit drugs more than three times in the last 30 days, sexual intercourse without a condom, involvement in fights with firearms and other types of weapons in the last 30 days, the adoption of unsafe practices in traffic (rarely or never using seatbelts in cars and helmets in motorcycles, or riding a vehicle with a drunk driver more than four times in the last 30 days), and driving without a permit more than four times in the last 30 days.

The variables of interest that were selected for the identification of potential policy approaches to minimize the co-occurrence of risk health behaviors among Brazilian adolescents were exposure to ≥1 health risk behavior, and coverage of the Family Health Strategy program at the municipal level. Other variables potentially linked to the exposure to and adoption of multiple risk behaviors included in the models were as follows:

- Individual characteristics: age bracket (<14 years old, 14–16 years old, or ≥17 years old); skin color/ethnicity (white, black, brown, yellow, or indigenous); gender (female or male); age-series distortion of educational attainment; frequency of consumption of foods considered healthy-eating markers (beans, fruits, and vegetables); frequency of consumption of foods considered unhealthy-eating markers (sweets, candies, and soft drinks); physical activity level within international recommendations.
- Household environment characteristics: socioeconomic strata (based on ownership of assets); living with one or two parents or other relatives; parents’ or relatives’ attitudes towards the youngster (attention to the adolescent’s leisure activities; frequency of family meals during the week; and allowing the adolescent to watch television or other activities during the meals).
- School environment characteristics: type of school (public or private); skipping school; relationship of the adolescent with colleagues (support from the colleagues; frequency of being a victim of bullying); security for participating in school activities (absences due to lack of security traveling to and from school; absences due to lack of security at school).
- Control variables: region of residence (North, Northeast, South, Southeast, or Midwest); year of the survey (2009, 2012, 2015, or 2019).

The categorization of individuals according to skin color/ethnicity into five standard categories officially adopted in Brazil (white, black, brown, yellow, or indigenous) was based on self-declared responses. Although quantitative studies usually report “yellow” skin color (East Asian descendants and admixed individuals reporting light yellow skin color) and “indigenous” ethnicity (native American individuals reporting indigenous ancestry) into a single group of “others”, the descriptive statistics of the present study presents them separately to promote the visibility of the groups [27].

In addition to data from the PeNSE, information from the Brazilian Ministry of Health on the coverage of the Family Health Strategy at the municipal level according to year was incorporated in the datasets to allow the identification of potential effects of the Brazilian Family Health Strategy on inequalities in the exposure and adoption of risk behaviors among adolescents.

### 2.3. Statistical Analyses

The descriptive statistics of individual, household, and school environment characteristics of individuals interviewed in the four editions of the survey were presented in frequency and 95% confidence intervals (discrete variables), or mean and standard error (continuous variables), according to year. The analyses were conducted using a complex survey design to allow representativeness at the population level.

The analysis of inequalities in health focused on the identification of differences in the occurrence of health outcomes among individuals in different socioeconomic levels through the estimation of concentration indexes, similar to the Gini index [28]. The concentration indexes measure the deviation between the situation of perfect equality and the current situation of the population, showing the cumulative proportion of individuals presenting the health outcome of interest within the population, ranked by socioeconomic level from the poorest to the richest.

The analyses of inequalities performed in the present study identify differences in the exposure to and adoption of multiple health risk behaviors among Brazilian students according to socioeconomic strata and year, allowing to verify changes in the proportion of adolescents declaring a co-occurrence of behaviors from 2009 to 2019. Thus, the concentration indexes estimated measure inequalities in the exposure to or adoption of multiple risk behaviors (*y*) according to the socioeconomic position of the adolescent in the population, showing the cumulative share of individuals exposed to or adopting multiple risk behaviors according to the cumulative proportion of individuals ranked from the lowest to the highest socioeconomic strata (Equation (1)).

(1) $$CI=\frac{2}{n\mu }\sum_{i=1}^{N}y_{i}R_{i}-1$$

where *µ* = mean of *y*; *R_i_* = proportional rank of the *i*th person in the income/wealth distribution; and *n* = sample size. Thus, it is possible to estimate the horizontal inequality index (*HI*) addressing the measurement of inequality across groups of individuals controlling for personal characteristics regarding the exposure to or adoption of multiple risk behaviors. The *HI* represents the difference between the overall concentration index (*CI*) and the concentration index linked to personal characteristics (*CN*) (Equation (2)).

(2) HI=CI−CN

In addition, the analyses of inequalities allow the disaggregation of the concentration indexes according to their potential determinants, using a set of independent variables linked to the exposure to or adoption of multiple risk behaviors. The share of socioeconomic inequalities attributable to individual characteristics accounts for potential sources of prejudice (gender, age, and skin color/ethnicity), and the share associated with external factors allows the identification of social pressures from the household and school environments.

The disaggregation of socioeconomic inequalities was performed through linear regression models, with the dependent variable representing the concentration index, y*, a latent non-observed variable describing the exposure or the adoption of multiple health risk behaviors (Equation (3)).

(3) $$y^{*}=\beta_{1}^{\prime }.X+\beta_{2}^{\prime }.Z+\varepsilon$$

where *β’_k_* = coefficient of the variable of interest *k*; *X* = matrix of variables referring to the individual characteristics (gender, age bracket, and skin color/ethnicity); *Z* = matrix of variables referring to the external factors (household environment, school environment, lifestyle choices, Family Health Strategy coverage, and geographical region); and *ε* = error term.

The descriptive statistics, and the estimation of concentration indexes and their disaggregation were performed using the software Stata, version 17.0, at a 5% statistical significance level, based on the application of survey sample weights through the utilization of a complex survey design that allows representativeness at the population level. Therefore, the characteristics of participants correspond to the distribution of students enrolled in public and private schools in Brazil during 2009, 2012, 2015, and 2019.

## 3. Results

Table 1 reports information on sociodemographic, household, and school characteristics, whereas Table 2 presents information on lifestyle characteristics, multiple health risk behavior indicators, and their components (health risk behaviors).

The majority of the participants in the survey were female (~54% in 2009 to ~51% in 2019) in the age bracket of 14–16 years old (~91% in 2009 to ~73% in 2019), self-declaring a white (~40% in 2009 to ~36% in 2019) or brown (~39% in 2009 to ~42% in 2019) skin color/ethnicity, from low socioeconomic strata (~41% in 2009 to ~47% in 2019), and living with both parents (~59% in 2009 to ~51% in 2019) in the Southeast region (~50% in 2009 to ~39% in 2019). The proportion of students presenting age-grade distortion showed a reduction during the decade (from 10.2% in 2009 to 9.4% in 2019) (Table 1).

A substantial proportion of the adolescents declared that their families regularly monitor their leisure activities (~57% in 2009 to ~71% in 2019), and frequently consume meals together (~79% in 2009 to ~67% in 2019). However, most of them also indicated that they frequently consume meals watching television (~57% in 2009 to ~73% in 2019). Regarding school environment, most students were enrolled in public schools (~79% in 2009 to 75% in 2019), and only a small proportion of adolescents declared absences at school due to a lack of security at the school (~5% in 2009 to ~11% in 2019), or on the way to the school (~6.2% in 2009 to ~13% in 2019). In addition, there was a high percentage of students declaring the frequent support of their colleagues at school (~66% in 2009 to ~65% in 2019), and skipping school (~18% in 2009 to ~21% in 2019). However, there was a substantial increase in the proportion of students being frequent victims of bullying (~5% in 2009 to ~22% in 2019). The Family Health Strategy program coverage increased until 2015 (~0.32 in 2009 to ~0.45 in 2015), and maintained a similar level in 2019 (~0.46) (Table 1).

Trends regarding the exposure to and adoption of multiple risk behaviors among adolescents in Brazil showed an increase in the proportion of adolescents declaring exposure to ≥1 health risk behavior (~91% in 2009 to ~96% in 2019) or ≥3 health risk behaviors (~35% in 2009 to ~48% in 2019), and the adoption of ≥2 health risk behaviors (~14% in 2009 to ~32% in 2019) (Table 2).

Yet, there were generally mixed patterns in the exposure to or adoption of isolated health risk behaviors. The proportion of adolescents reporting involvement in fights with firearms and/or other weapons showed a decrease during the period from 2009 to 2019, whereas the proportion of students declaring an experience and regular use of illicit drugs, sexual intercourse and unsafe sex, an abusive consumption of alcoholic beverages, and dangerous traffic experiences and practices presented a statistically significant increase (Table 2).

The exposure to and adoption of risk behaviors presented a significant association with the major part of the personal, household, and school characteristics during the period of the study (Table 3 and Table 4). The exceptions were the association of the exposure to and adoption of risk behaviors among adolescents in the North region of Brazil in 2019, declaring white skin color/ethnicity and being enrolled in a public school in 2009, and being in a family of low social class in 2015.

**Figure 1:**
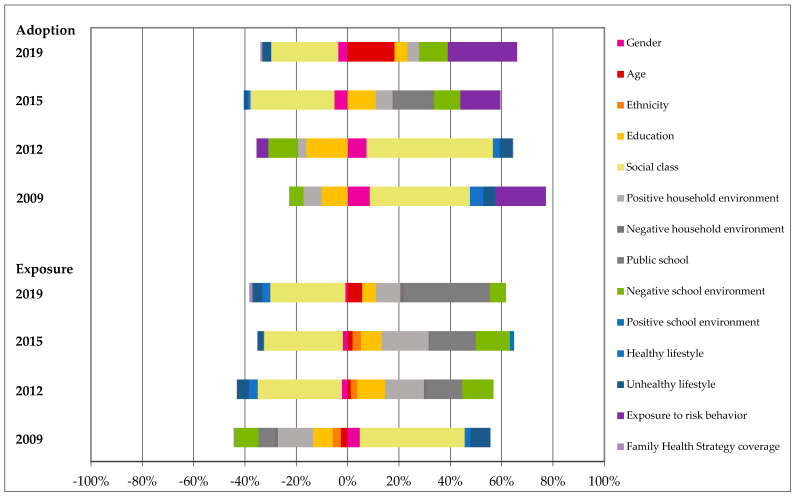
Disaggregation of concentration indexes for exposure to and adoption of risk behaviors according to the contribution of determinants. Brazil, 2009–2019.

Table 5 and Figure 1 present data on the concentration indexes (i.e., socioeconomic inequality in the incidence of multiple health risk behaviors), horizontal inequalities (i.e., CI excluding inequalities attributable to personal characteristics like gender, age, and skin color/ethnicity), and the coefficients representing the effects of major determinants of socioeconomic inequalities (i.e., their contribution to the scenario of inequality).

In addition to the increase in the incidence of the exposure to and adoption of multiple risk behaviors among Brazilian youngsters between 2009 to 2019, the concentration indexes showed an increase in inequalities referring to the co-occurrence of health risk behaviors throughout the period of analysis. The concentration indexes for the exposure (0.034) to and adoption (0.084) of health risk behaviors in 2009 indicate a higher incidence among adolescents from a high socioeconomic status, followed by a transition to negative concentration indexes for the exposure to and adoption of risk behaviors in 2012 and 2015, respectively (Table 5).

The estimation of the horizontal inequality index showed similar trends, presenting a similar magnitude to the concentration index, which indicates that socioeconomic inequalities in the exposure to and adoption of health risk behaviors were generally linked to external factors, i.e., the influence of household and school environments surrounding adolescents (Table 5).

The disaggregation of socioeconomic inequalities into major groups of determinants indicated that socioeconomic strata represented the main factor contributing to the occurrence of differences in the exposure (0.055 in 2009 to 0.040 in 2019) to and adoption (0.049 in 2009 to 0.019 in 2019) of multiple health risk behaviors among Brazilian youngsters. In addition, a positive household environment (−0.018 in 2009 to −0.013 in 2019) influenced the exposure to multiple health risk behaviors, whereas enrollment in public schools and a negative school environment presented effects in both the exposure to and adoption of multiple health risk behaviors.

Nevertheless, the Family Health Strategy coverage at the municipal level presented very low coefficients (although statistically significant), i.e., indicating the minor role of the public policy of health in influencing socioeconomic inequalities in multiple health risk behaviors. Early exposure to any health risk behavior showed a higher influence on socioeconomic inequalities in the adoption of multiple risk behaviors than the coverage of the Family Health Strategy (Table 5).

Figure 1 presents the proportional contribution of the main groups of determinants of socioeconomic inequalities to the concentration indexes. The positive concentration indexes, representing a higher incidence of multiple health risk behaviors among wealthier individuals, present a distribution of determinants skewed to the positive effects (right side of the graph) for the exposure to and adoption of risk behaviors in 2009, changing to negative concentration indexes for the exposure to multiple risk behaviors in 2012, and for the adoption of multiple risk behaviors in 2015.

## 4. Discussion

The present study investigated the evolution and determinants of socioeconomic inequalities in the exposure to and adoption of multiple risk behaviors among Brazilian adolescents between 2009 and 2019. The results showed an intensification in socioeconomic inequalities referring to the incidence of multiple risk behaviors, shifting from a higher concentration among wealthier individuals in 2009 to lower socioeconomic strata in the following periods. The determinants of inequalities in the exposure to and adoption of multiple risk behaviors among Brazilian adolescents were predominantly connected to socioeconomic status, enrollment in public school, positive household environments, and negative school environments.

Individuals in the transition from childhood to adolescence are susceptible to adoption risk behaviors due to neural reward sensitivity, hindering the effects of cognitive performance. Certain rewards and social contexts may intensify the activation or the connectivity of the affective-motivational system in youngsters, increasing the probability of risky behaviors and the influence of peers [29,30]. Changes in the individual’s behaviors may be difficult to achieve without addressing the social context, considering that diverse determinants may influence the exposure to and adoption of risk behaviors, including individual characteristics, and household and school environments, in addition to incentives of public policies, economic scenarios, and others [31,32].

The results showed that Brazilian adolescents generally live in positive household environments marked by the constant supervision of adolescents’ leisure activities by their parents and relatives, and frequent family meals during the week. However, the proportion of adolescents reporting the consumption of meals watching television and performing other tasks increased throughout the period of study. Concerning school environments, Brazilian students usually reported positive features (e.g., support from colleagues, and a feeling of security at school), although there was growth in the proportion of participants declaring bullying victimization and absence from school due to insecurity.

The results indicated a lower exposure to and adoption of multiple health risk behaviors among younger female teenagers living with both parents in positive household and school environments. Accordingly, other studies showed that older male adolescents experiencing family disruption were susceptible to the adoption of certain risk behaviors [33,34]. In addition, the incidence of multiple health risk behaviors was ~35% to ~40% among Brazilian students between 2009 and 2015, similar to the patterns identified in the study investigating data from the Global School-Based Student Health Survey from 2007 to 2016, which showed that approximately one-third of students (34.9%) reported more than two risk factors, with males (36.3%) being more susceptible than females (33.5%). The prevalence of ≥3 risk factors increased according to age, being 29.4% among youngsters between 11 and 13 years old, and 46.4% among adolescents between 16 and 17 years old [35].

The findings of the present study showed a high exposure to and adoption of health risk behaviors related to the consumption of alcoholic beverages among Brazilian adolescents from 2009 to 2019. Furthermore, the proportion of youngsters reporting abusive alcohol consumption significantly increased throughout the period. Children and adolescents tend to initiate the consumption of alcoholic beverages through socialization with older individuals [36,37,38], and early experience with alcohol consumption increases the probability of alcohol dependence [39].

In addition, there was an increase in the incidence of risk behaviors related to driving without a permit, and unsafe traffic practices like the lack of the use of seatbelts in cars, riding motorcycles without a helmet, and being in vehicles driven by drunk drivers were also present among students interviewed in Brazilian schools. Recent evidence indicates an association between sensation-seeking among youngsters and road safety intentions, attitudes, and behaviors [40]. Sensation-seeking results in a higher propensity for the occurrence of road traffic injuries, especially in combination with alcohol consumption [33,34].

The results of the present study also pointed to a significant growth in early sexual initiation among adolescents, particularly the adoption of unsafe sex practices among Brazilian students between 2009 and 2019. Approximately 28% of Brazilian students experienced sexual relations between 2009 and 2019. The findings are consistent with data from the Brazilian Ministry of Health [41], showing that Brazilian boys usually start sexual experiences at 12 years and 9 months, whilst Brazilian girls tend to experience sex at 13 years and 7 months. However, the increasing adoption of risky sexual behavior among Brazilian students throughout the period differs from the results of a study with German adolescents [42]. Other studies present mixed evidence on the adoption of risky sexual behavior among youngsters, being associated with earlier sexual initiation in Australia [43], and older adolescent males in Canada [44], showing that sexual experiences comprise complex behaviors influenced by diverse personal, familial, and sociocultural factors [45].

Risk behaviors linked to tobacco use decreased in Brazil between 2009 and 2019. The reduction in tobacco use has been the focus of numerous public health campaigns in Brazil in recent decades. Furthermore, early smoking initiation increases the probability of the occurrence of chronic diseases and premature mortality [46]. In Brazil, specific legislation prohibits sales of tobacco products to individuals ≤18 years old. However, approximately 1.3 million adolescents aged 12 to 17 have already experienced cigarettes in their lifetime, an incidence of approximately 34% of the population [39], which was higher than the incidence identified in the present study.

The results of the present study also showed a significant rise in the occurrence of the exposure to and adoption of health risk behaviors linked to illicit drug utilization among Brazilian adolescents in the period from 2009 to 2019. A previous study indicated that experiences with drugs tend to occur among young individuals in the United States in 2019 [47], and the present findings indicate that the proportion of youngsters experiencing and frequently consuming illicit drugs has increased throughout the period of analysis.

Regarding interpersonal violence, it is important to highlight that Brazil is one of the countries with the highest mortality rates due to firearms and other weapons, particularly among adolescents and young adults [48]. Higher levels of violence have been reported in the literature among older male adolescents in minority groups (particularly black and brown skin color/ethnicity) in Brazil between 2001 and 2017 [49].

The analysis of inequalities showed that the exposure to and adoption of multiple health risk behaviors were significantly associated with socioeconomic strata, a positive household environment, and a negative school environment. Our findings corroborate previous evidence on the connections between the coexistence of multiple risk behaviors and the family and school contexts of youngsters [12,32,49,50,51,52,53].

Positive household environments, represented by the involvement of family members in child development (e.g., the family monitoring of leisure activities and the consumption of family meals) showed important effects on the inequalities related to the exposure to and adoption of multiple health risk behaviors between 2009 and 2019. Family support may include intermediate effects through the adoption of healthy lifestyle patterns (e.g., incentives to higher frequency in the consumption of fruits, vegetables and beans, and lower frequency in the consumption of candies, sweets, and soft drinks), according to the evidence from a previous study with Brazilian students [53]. Parental mediation theory indicates that the role of parents may mitigate the influence of others on children’s and adolescents’ behavior, reducing exposure and adherence to multiple health risk behaviors [54].

Concerning the school environment, negative experiences were associated with an increase in inequalities in the exposure to and adoption of multiple health risk behaviors among Brazilian students. Students who reported being victims of bullying by school colleagues, being absent from school due to a lack of security at school and on the way to school, and adolescents enrolled in public schools were more likely to engage in multiple risk behaviors. Previous evidence showed that adolescents experiencing school bullying victimization in the United States presented higher odds of suicidal ideation, in addition to a higher occurrence of suicidal ideation associated with alcohol abuse and illicit drug utilization [49].

There were increasing socioeconomic inequalities in the exposure to and adoption of multiple risk behaviors among Brazilian students throughout the period from 2009 to 2019. Furthermore, the coverage of the Family Health Strategy at the municipal level showed minor effects on socioeconomic inequalities in multiple health risk factors throughout the periods in Brazil. Yet, it is important to emphasize the lack of evidence in the literature regarding the role of public policies of health on socioeconomic inequalities related to multiple risk behaviors among youngsters in Brazil.

The only study investigating trends in socioeconomic inequalities related to health behaviors among Brazilian students focused on the isolated occurrence of certain risk behaviors, identifying a decrease in inequality referring to alcohol consumption, and an increase in inequality regarding behaviors associated with violence (domestic violence, fights with guns, and bullying) from 2009 to 2012 [18]. Nonetheless, the study from Azeredo et al. [18] presented limitations in the analysis of data, lacking an identification of determinants of inequalities in the exposure to and adoption of risk behaviors.

Therefore, the evidence in the present study contributes to the field by presenting and investigating the differences between the exposure to and adoption of multiple risk behaviors among youngsters throughout the 10 years from 2009 to 2019. There was an intensification of socioeconomic inequalities in the exposure to and adoption of multiple risk behaviors among students in Brazil, predominantly attributable to the effects of a positive household environment and a negative school environment, in addition to the effects of lifestyle choices [55].

Enrollment in public schools also presented important effects on socioeconomic inequalities in the exposure to and adoption of multiple risk behaviors among adolescents in Brazil. Public schools are financed by the government to provide education without charges to students or parents in Brazil; therefore, various studies explore the role of public schools on educational attainment, the quality of education, health behaviors, and nutrition. A previous study showed the influence of enrollment in public schools on adolescents’ adoption of risk behaviors linked to unhealthy practices of weight control [56]. In addition to the factors identified in the study, changes in socioeconomic inequality regarding multiple risk behaviors were probably associated with changes in the economic situation of the country [57]. Families usually transfer their children from private to public schools during economic crises in Brazil; therefore, the influence of public schools on socioeconomic inequalities changes throughout time depending on the current contextual scenario [58].

This study has certain limitations. First, the study was based on the analysis of data from the National Student Health Survey (PeNSE), designed to comprise a system to monitor students’ exposure to health risk and protection factors [18]. Thus, considering that the Brazilian survey has a cross-sectional design, the analyses lack conditions to establish causal relationships between risk behaviors and individual, environmental, and lifestyle characteristics.

Second, the information in the four editions of the survey was obtained through self-administered questionnaires, which may result in errors due to the misinterpretation of questions, underreporting, or a lack of response. Third, the changes introduced in the questionnaire throughout the four editions of the survey also represented an additional challenge for the analysis through the comparison of information across years, limiting the information available for the analyses. Fourth, the survey lacked information on students’ religion, which may be an additional source of influence on adolescents’ decisions in relation to their adherence to multiple health risk behaviors [59].

However, the selection of variables that were directly comparable throughout the four editions in the present study allowed the researchers to maintain consistency in the analysis for the estimation of models encompassing data from 2009 to 2019. Furthermore, the utilization of data representative at the population level provides robustness to the statistical analyses conducted, resulting in solid evidence that may support evidence-based decision-making processes in public policies of health in Brazil.

Finally, it is important to highlight that the sample represents individuals enrolled in Brazilian schools, and therefore, adolescents without enrollment in schools were excluded from the sample. Thus, despite comprising a methodology generally adopted in several countries due to the accessibility to the target population, school-based surveys neglect vulnerable individuals without access to schools. Yet, it is important to acknowledge that, since the 1980s, the population coverage of the Brazilian education system showed a substantial increase, approaching universality. Considering that the four editions of the survey were conducted in public and private schools, the data provide adequate representativeness of the target population at the national level.

## 5. Conclusions

The findings of the present study highlight the complex mechanisms involved in the exposure to and adoption of multiple risk behaviors during the transition into adolescence, including trends and determinants of inequalities between 2009 and 2019. There was a significant increase in inequalities in the exposure to and adoption of simultaneous risk behaviors among students in this period, which was concentrated among individuals in higher social strata. However, older male adolescents in minority groups (black and brown individuals) with lower educational attainment (repeat students) presented an increased probability of exposure to and adoption of multiple risk behaviors.

Contextual issues referring to lifestyle choices, household, and school environments showed an important role in the occurrence of inequalities towards adolescents’ exposure to or adoption of multiple risk behaviors. Therefore, public health interventions for adolescents should focus on specific strategies based on the prevention of risky behaviors among youngsters through social assistance and school-based activities, particularly in public schools, to avoid the onset of negative health outcomes. In addition, programs targeting health education directed at households with lower socioeconomic status may comprise evidence-based strategies to reduce the exposure risk behaviors during critical life stages, targeting early intervention based on comprehensive support for the engagement of children and adolescents in activities designed to incentivize healthy behaviors at school and during leisure.

## Figures and Tables

**Table 1:** Sociodemographic and environmental characteristics of adolescents. Brazil, 2009–2019.

Sociodemographic Characteristics	2009		2012		2015		2019	
N	63,411		61,145		51,135		40,017	
	%	95%CI	%	95%CI	%	95%CI	%	95%CI
Gender								
Male	46.2	45.3;47.0	48.8	48.0;49.6	48.8	47.9;49.7	49.5	48.3;50.7
Female	53.8	53.0;54.7	51.2	50.4;52.0	51.2	50.3;52.1	50.5	49.3;51.7
Age								
<14 years old	2.3	2.1;2.6	2.4	2.2;2.7	1.5	1.3;1.7	0.2	0.2;0.3
14–16 years old	90.9	90.1;91.6	90.9	90.1;91.5	93.1	92.5;93.6	72.6	69.9;75.1
≥17 years old	6.8	6.1;7.5	6.7	6.1;7.4	5.5	4.9;6.0	27.2	24.6;29.8
Skin color/Ethnicity								
White	40.4	38.6;42.2	37.8	36.2;39.5	36.7	34.9;38.5	35.8	33.4;38.3
Black	12.6	11.8;13.5	14.0	13.3;14.8	13.3	12.6;14.1	15.1	13.9;16.4
Brown	39.2	37.7;40.7	40.0	38.7;41.4	41.8	40.2;43.3	42.3	40.1;44.5
Yellow	3.7	3.4;4.1	4.5	4.2;4.8	5.1	4.7;5.4	3.7	3.4;4.2
Indigenous	4.0	3.7;4.4	3.7	3.4;3.9	3.2	2.9;3.4	3.0	2.6;3.5
Age-grade distortion	10.2	9.3;11.2	11.4	10.5;12.4	8.6	7.9;9.4	9.4	8.0;10.9
Family Health Strategy coverage ^1^	0.32	0.31;0.33	0.38	0.38;0.39	0.45	0.44;0.46	0.46	0.44;0.47
Region								
North	10.6	9.1;12.4	11.6	10.2;13.3	12.9	11.3;14.6	15.6	10.6;22.5
Northeast	23.5	20.9;26.4	23.8	21.5;26.3	24.0	21.6;26.5	24.1	19.8;28.9
Southeast	49.8	45.3;54.3	45.2	41.1;49.3	44.5	40.4;48.6	39.2	32.0;46.8
South	5.9	5.0;7.1	7.1	6.0;8.4	6.0	5.0;7.1	7.6	5.6;10.4
Middle-West	10.1	8.7;11.7	12.2	10.7;14.0	12.7	11.1;14.6	13.5	10.0;18.0
Household characteristics	2009		2012		2015		2019	
Socioeconomic strata								
Low	41.1	39.0;43.3	32.7	31.0;34.5	47.6	45.4;49.8	47.2	44.7;49.7
Middle	47.5	45.7;49.2	56.4	55.1;57.7	46.5	44.8;48.3	47.3	45.4;49.2
High	11.4	9.7;13.5	10.9	9.5;12.4	5.9	4.7;7.3	5.5	4.5;6.8
Living with parents	59.3	58.1;60.4	56.8	55.7;57.8	55.8	54.6;57.0	51.0	49.7;52.2
Family monitoring leisure activities	57.0	55.8;58.2	60.5	59.5;61.5	67.6	66.5;68.6	70.6	69.5;71.6
Consumption of meals with family	69.6	68.8;70.4	67.9	67.0;68.8	74.0	73.2;74.8	66.5	65.2;67.7
Consumption of meals watching TV	56.5	55.5;57.5	59.4	58.5;60.2	55.9	54.9;56.9	72.8	71.2;74.3
School characteristics	2009		2012		2015		2019	
Type of school								
Private	20.9	17.8;24.4	25.6	22.5;29.0	27.6	24.3;31.3	25.0	20.9;29.7
Public	79.1	75.6;82.2	74.4	71.0;77.5	72.4	68.7;75.7	75.0	70.3;79.1
Skipping school	18.2	17.2;19.2	25.6	24.5;26.8	23.6	22.5;24.7	21.4	19.9;22.9
Support of colleagues in school	65.5	64.2;66.7	61.8	60.6;63.0	65.0	63.9;66.0	64.9	63.5;66.3
Victim of bullying	4.6	4.2;5.1	16.2	15.7;16.8	21.6	20.9;22.2	21.5	20.3;22.6
Lack of security at school	5.2	4.7;5.8	7.9	7.3;8.5	9.1	8.5;9.8	11.4	10.5;12.4
Lack of security on the way to school	6.2	5.7;6.8	8.9	8.3;9.6	12.6	11.9;13.4	13.4	12.3;14.5

Figures express frequencies. 95% CI, 95% confidence interval. ^1^ mean ± standard deviation. Analyses include sample weight and complex survey design for population-level representativeness.

**Table 2:** Lifestyle characteristics and risk behaviors of adolescents. Brazil, 2009–2019.

Lifestyle Characteristics	2009		2012		2015		2019	
N	63,411		61,145		51,135		40,017	
	%	95%CI	%	95%CI	%	95%CI	%	95%CI
Recommended physical activity level	23.4	22.5;24.3	34.1	33.2;35.0	36.5	35.6;37.5	28.9	27.9;30.0
Healthy food consumption pattern ^1^	0.52	0.51;0.52	0.53	0.52;0.54	0.54	0.53;0.54	0.48	0.47;0.49
Unhealthy food consumption pattern ^1^	0.56	0.55;0.57	0.53	0.52;0.53	0.49	0.49;0.5	0.43	0.43;0.44
Risk behaviors	2009		2012		2015		2019	
Exposure to ≥1 risk behavior	91.3	90.7;91.8	93.5	93.0;93.9	95.2	94.8;95.5	96.2	95.7;96.6
Exposure to ≥3 risk behaviors	34.9	33.7;36.1	39.7	38.6;40.8	36.7	35.4;38.0	48.0	46.3;49.7
Use of cigarettes	21.5	20.5;22.6	21.7	20.8;22.6	18.7	17.9;19.6	26.4	25.2;27.6
Consumption of alcohol	68.3	67.4;69.2	62.1	61.2;63.0	54.4	53.3;55.6	70.3	68.9;71.6
Use of illicit drugs	7.6	7.0;8.2	9.3	8.6;10.0	10.2	9.6;10.9	17.7	16.5;19
Sexual experience	27.3	26.1;28.6	30.4	29.3;31.6	26.7	25.5;28.1	39.6	37.7;41.5
Involvement in fight with firearms	3.5	3.2;3.9	6.6	6.2;6.9	5.4	5.0;5.8	2.5	2.1;2.9
Involvement in fight with other weapons	5.4	5.0;5.8	7.8	7.3;8.3	7.9	7.5;8.4	4.3	3.8;4.8
Driving without permit	17.9	17.1;18.7	22.3	21.5;23.0	24.6	23.7;25.6	24.2	23.2;25.2
Adoption of unsafe traffic practices	72.0	70.7;73.3	80.9	79.7;81.9	87.2	86.4;87.9	87.2	86.1;88.2
Adoption of ≥2 behaviors	14.2	13.5;14.8	18.9	18.2;19.7	20.7	19.8;21.7	31.5	30.0;33.0
Use of cigarettes ≥ 6 days in last 30 days	2.1	1.8;2.4	2.0	1.8;2.3	1.7	1.5;1.9	2.7	2.4;3.0
Abusive alcohol consumption ≥ 3 times in life	6.0	5.6;6.4	8.0	7.5;8.5	7.4	6.9;7.9	16.8	15.8;17.9
Use of illicit drugs ≥ 3 times in last 30 days	1.2	1.0;1.4	1.7	1.5;2.0	2.3	2.1;2.6	3.6	3.1;4.1
Sexual intercourse without condom	6.2	5.7;6.7	7.7	7.3;8.2	9.7	9.0;10.4	17.5	16.2;18.8
Involvement in fights with weapons	2.2	2.0;2.5	3.5	3.3;3.8	3.3	3.0;3.6	1.6	1.3;1.8
Frequent unsafe traffic practices	7.9	7.4;8.4	9.7	9.2;10.3	10.6	10.1;11.2	12.1	11.2;12.9

Figures express frequencies. 95% CI, 95% confidence interval. ^1^ mean ± standard deviation. Analyses include sample weight and complex survey design for population-level representativeness.

**Table 3:** Associations between exposure to risk behaviors in relation to personal, household and school characteristics. Brazil, 2009–2019.

Characteristics		Exposure to ≥3 Risk Behaviors							
		2009		2012		2015		2019	
		**%**	**Sig.**	**%**	**Sig.**	**%**	**Sig.**	**%**	**Sig.**
Gender	Male	44.69	<0.001	47.73	<0.001	43.27	<0.001	52.49	<0.001
	Female	28.54		33.78		30.36		43.65	
Age	<14 years old	17.60	<0.001	22.75	<0.001	15.59	<0.001	28.38	<0.001
	14–16 years old	34.87		39.09		35.16		40.85	
	≥17 years old	59.88		67.28		68.85		67.34	
Ethnicity	White	34.47	<0.001	37.21	<0.001	33.16	<0.001	43.74	<0.001
	Others	37.45		42.71		38.80		50.48	
Age-grade distortion	Yes	33.62	<0.001	37.18	<0.001	33.83	<0.001	45.35	<0.001
	No	58.39		67.26		66.83		73.98	
Social class	Low	33.67	<0.001	39.82	<0.001	37.83	0.001	50.89	<0.001
	Middle	38.33		42.05		36.36		46.28	
	High	36.66		35.72		30.30		39.29	
Living with parents	Yes	32.47	<0.001	35.95	<0.001	31.88	<0.001	40.94	<0.001
	No	41.43		46.69		42.86		55.47	
Region	North	35.40	<0.001	42.90	<0.001	41.11	<0.001	49.89	0.174
	Northeast	34.02		37.81		34.01		46.81	
	Southeast	36.17		39.95		34.92		46.81	
	South	42.03		45.01		42.16		50.55	
	Middle-West	38.31		43.88		41.03		50.68	
Family monitoring leisure activities	Yes	30.76	<0.001	33.26	<0.001	29.86	<0.001	43.41	<0.001
	No	42.60		51.36		51.06		58.95	
Consumption of meals with family	Yes	34.42	<0.001	37.82	<0.001	34.24	<0.001	44.46	<0.001
	No	39.76		46.24		43.93		55.27	
Consumption of meals watching TV	Yes	39.58	<0.001	44.10	<0.001	39.88	<0.001	51.02	<0.001
	No	31.29		35.24		32.82		40.47	
Public school	Yes	37.04	<0.001	43.44	<0.001	40.13	<0.001	52.12	<0.001
	No	33.03		32.43		27.52		35.79	
Skipping school	Yes	56.77	<0.001	58.59	<0.001	53.83	<0.001	66.13	<0.001
	No	31.32		34.22		31.52		43.14	
Support of colleagues in school	Yes	34.36	<0.001	38.38	<0.001	33.88	<0.001	45.19	<0.001
	No	39.04		43.89		42.14		53.32	
Victim of bullying	Yes	66.54	<0.001	43.05	0.003	39.53	<0.001	53.72	<0.001
	No	34.46		39.99		35.99		46.65	
Lack of security at school	Yes	53.96	<0.001	59.06	<0.001	58.89	<0.001	63.10	<0.001
	No	35.03		38.82		34.52		46.09	
Lack of security on the way to school	Yes	52.89	<0.001	56.62	<0.001	53.76	<0.001	63.80	<0.001
	No	34.94		38.91		34.32		45.62	

Figures express frequencies. Analyses include sample weight and complex survey design for population-level representativeness.

**Table 4:** Associations between adoption of risk behaviors in relation to personal, household and school characteristics. Brazil, 2009–2019.

Characteristics		Adoption of ≥2 Risk Behaviors							
		2009		2012		2015		2019	
		%	Sig.	%	Sig.	%	Sig.	%	Sig.
Gender	Male	21.67	<0.001	25.74	<0.001	27.21	<0.001	35.79	<0.001
	Female	9.56		13.26		14.30		27.40	
Age	<14 years old	8.59	<0.001	11.04	<0.001	9.42	<0.001	30.12	<0.001
	14–16 years old	14.19		18.21		19.47		25.20	
	≥17 years old	31.35		37.97		43.90		48.43	
Ethnicity	White	14.80	0.097	18.04	<0.001	18.76	<0.001	28.81	<0.001
	Others	15.64		20.23		21.77		33.22	
Age-grade distortion	Yes	13.51	<0.001	16.92	<0.001	18.51	<0.001	29.06	<0.001
	No	30.35		38.49		43.09		55.59	
Social class	Low	12.95	<0.001	17.47	<0.001	20.96	0.572	32.73	0.032
	Middle	16.85		20.69		20.47		30.73	
	High	17.48		18.54		19.51		29.52	
Living with parents	Yes	14.03	<0.001	17.33	<0.001	18.20	<0.001	26.40	<0.001
	No	16.95		22.07		23.80		36.98	
Region	North	14.91	<0.001	19.37	0.004	22.28	0.003	29.47	0.594
	Northeast	14.33		18.69		20.65		31.99	
	Southeast	14.98		18.83		19.19		31.84	
	South	18.01		20.63		22.15		32.91	
	Middle-West	17.54		22.15		23.44		32.10	
Family monitoring leisure activities	Yes	11.87	<0.001	15.44	<0.001	16.15	<0.001	28.09	<0.001
	No	18.90		25.04		30.04		39.61	
Consumption of meals with family	Yes	14.22	<0.001	18.07	<0.001	19.61	<0.001	28.64	<0.001
	No	17.02		21.84		23.69		37.48	
Consumption of meals watching TV	Yes	16.69	<0.001	21.30	<0.001	23.01	<0.001	33.72	<0.001
	No	12.91		16.28		17.75		26.08	
Public school	Yes	15.52	0.085	20.42	<0.001	22.71	<0.001	34.28	<0.001
	No	14.50		16.42		15.13		23.48	
Skipping school	Yes	26.06	<0.001	29.80	<0.001	31.51	<0.001	48.10	<0.001
	No	12.49		15.59		17.33		27.01	
Support of colleagues in school	Yes	13.99	<0.001	18.20	<0.001	18.83	<0.001	29.33	<0.001
	No	16.79		21.00		24.19		35.49	
Victim of bullying	Yes	38.41	<0.001	21.48	0.003	22.58	0.002	34.33	0.004
	No	13.80		18.85		20.19		30.88	
Lack of security at school	Yes	28.85	<0.001	34.03	<0.001	37.54	<0.001	44.35	<0.001
	No	14.23		17.97		19.00		29.82	
Lack of security on the way to school	Yes	26.95	<0.001	30.30	<0.001	33.63	<0.001	45.18	<0.001
	No	14.18		18.20		18.85		29.45	

Figures express frequencies. Analyses include sample weight and complex survey design for population-level representativeness.

**Table 5:** Trends in inequalities in exposure to and adoption of multiple risk behaviors among adolescents. Brazil, 2009–2019.

Exposure to Risk Behavior	2009	2012	2015	2019
CI	0.034	−0.006	−0.021	−0.032
HI	0.036	−0.004	−0.017	−0.026
Gender	0.006	0.003	0.002	0.001
Age	−0.004	−0.002	−0.002	−0.008
Skin color/Ethnicity	−0.004	−0.003	−0.004	0.000
Age-grade distortion	−0.010	−0.013	−0.010	−0.008
Socioeconomic strata	0.055	0.040	0.038	0.040
Positive household environment	−0.018	−0.019	−0.022	−0.013
Negative household environment	−0.002	−0.002	−0.001	−0.002
Public school	−0.009	−0.017	−0.022	−0.046
Positive school environment	0.000	0.000	−0.002	0.000
Negative school environment	−0.013	−0.015	−0.016	−0.008
Healthy lifestyle patterns	0.003	0.004	0.001	0.004
Unhealthy lifestyle patterns	0.010	0.006	0.003	0.005
Family Health Strategy coverage	0.000	0.000	0.000	0.002
Region	0.004	0.003	0.003	0.000
Residual	0.016	0.008	0.012	0.000
Adoption of Risk Behavior	2009	2012	2015	2019
CI	0.084	0.033	−0.004	−0.018
HI	0.073	0.028	−0.009	−0.008
Gender	0.011	0.006	0.004	0.003
Age	0.000	0.000	0.000	−0.013
Skin color/Ethnicity	0.000	0.000	0.000	0.000
Age-grade distortion	−0.013	−0.012	−0.009	−0.004
Socioeconomic strata	0.049	0.037	0.026	0.019
Positive household environment	−0.009	−0.002	−0.005	−0.003
Negative household environment	0.000	0.000	−0.001	0.000
Public school	0.000	0.000	−0.012	0.000
Positive school environment	0.000	0.000	0.000	0.000
Negative school environment	−0.007	−0.009	−0.008	−0.008
Healthy lifestyle patterns	0.007	0.002	0.001	0.000
Unhealthy lifestyle patterns	0.006	0.004	0.001	0.003
Exposure to risk behavior	0.025	−0.003	−0.013	−0.020
Family Health Strategy coverage	0.000	0.000	0.000	0.001
Region	−0.002	−0.001	0.000	0.001
Residual	0.017	0.012	0.011	0.004

CI = concentration index; HI = horizontal inequality index. Analyses include sample weight and complex survey design for population-level representativeness. Coefficients shown refer to statistically significant determinants of concentration indexes.

## Data Availability

The study analyzed publicly available datasets that may be accessed in the public domain of the Brazilian Institute for Geography and Statistics (IBGE): https://www.ibge.gov.br/estatisticas/sociais/saude/9134-pesquisa-nacional-de-saude-do-escolar.html?=&t=microdados (accessed 10 October 2023).
